# Northerner *v*. Southerner

**Published:** 1890-12-20

**Authors:** 


### Editor's Letter-Box.

[Our Correspondents are reminded that prolixity is a great bar to publication, and that brevity of style and conciseness of statement greatly facilitate early insertion.]

### NORTHERNER *v.* SOUTHERNER.

"A Yorkshire woman" writes: I think "An Englishman" has mistaken the meaning of the reviewer of Mr. Besant's "Captain Cook." He assumes it to be that "firmness of purpose, taciturnity, and self-reliance" are a monopoly of the North country people. I do not for a moment suppose that he means to insinuate the Southerners are deficient in these qualities, only that the Northerners possess them to a marked extent, and in this view every one who knows Yorkshire people will cordially agree. Firmness of purpose is possessed by them often to an alarming extent, amounting perhaps to obstinacy, or a Southerner might term it pig-headedness, and in his determination to accomplish his purpose, a Northerner is often oblivious of anything beyond that purpose. I consider Captain Cook a typical Yorkshireman, and we see how in his firmness of purpose the savages and their customs were not considered; no doubt, had he exercised kindness and tact he would have lived to command other expeditions. When some virtues are possessed in an abnormal degree, there must, in our human economy, be a deficiency of others, and I think in the exercise of tact and *savoir faire*, the Southerners as a people, are superior. "An Englishman" declines to discuss "taciturnity," and though "suffering in silence," may not be Dr. Johnson's meaning of the word, still that virtue (which Captain Cook appears to have possessed strongly) is not typical of the Southerners who are always more ready to air their grievances than the Northerners. I have lived several years south of London, and I think whilst remembering we are all Englishmen, as your correspondent implies, we may with advantage learn from one another; but I do not see that any comparison was intended to be drawn.

